# High-moisture extrusion cooking on soybean-wheat protein mixtures: Effect of sodium alginate/xanthan gum/maltodextrin on promoting a fibrous structure

**DOI:** 10.3389/fnut.2022.1077601

**Published:** 2023-01-09

**Authors:** Fengqiujie Wang, Yang Gao, Xuelian Gu, Binyu Luan, Ying Zhu, Yuyang Huang, Xiuqing Zhu

**Affiliations:** College of Food Engineering, Harbin University of Commerce, Harbin, Heilongjiang, China

**Keywords:** high-moisture extrusion cooking, soybean protein, wheat protein, polysaccharide, dead-stop operation

## Abstract

At present, the changes in fibrous structure of plant proteins improved by polysaccharides during high-moisture extrusion cooking (HMEC) are still unclear. In this study, different additions (1, 2, 3, 4, and 5%) of sodium alginate (SA), xanthan gum (XG), and maltodextrin (MD) were used in the preparation of organised protein products based on soybean protein and wheat protein under high moisture extrusion conditions. It was revealed that SA-4%, XG-2%, and MD-2% (w/w) significantly enhanced the structural and physical properties of the fibres. The polysaccharides increased the water distribution of extrudates by enhancing protein-water interactions through hydrogen bonding, with MD-2% having the strongest ability to trap free water. The mechanism by which the polysaccharides improved the fibrous structure of extrudates involved the reorganization of molten proteins from the die head region to the cooling region, formation of new molecular bonds and enhancement of thermal stability. XG-2% significantly increased the β-sheet structure in the molten region (48.9 ± 1.35%) and showed the best thermal stability. Overall, SA-4% was able to better maintain the molecular bonding transformation and strong water absorption, which stabilised the protein conformation and formed the highest fibrous degree (2.1 ± 0.03). This suggests that the properties of the three polysaccharides can be used as modifiers of high water extruded plant proteins to improve the extruded materiality, functional and nutritional properties.

## Graphical Abstract

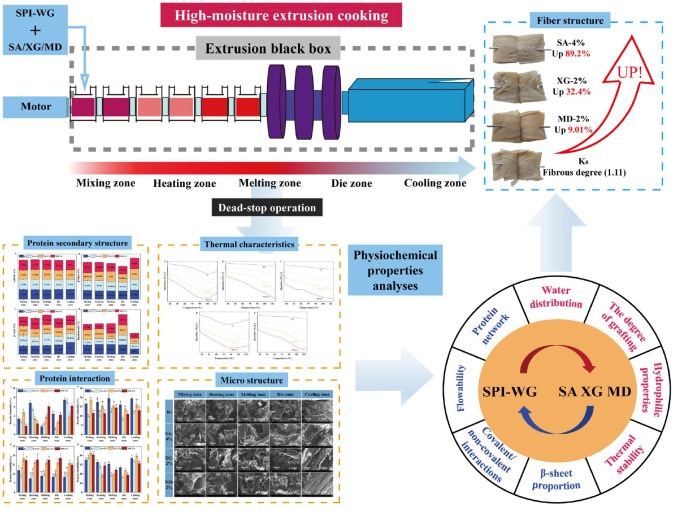

## 1. Introduction

With the increased demand for supply in the meat consumption market and the reduction of available land for livestock farming, plant-based products have been developed and are gradually being used as a nutritious alternative to animal protein (1, 2). In particular, High-moisture extrusion cooking of plant-based products is an important green processing technology for building healthy ecology and addressing food safety (3, 4). This can be attributed to the high-moisture extrusion cooking process of plant protein, in which the protein is subjected to the interaction of high-temperature heating, high pressure, strong shear forces, and large amounts of water (40–80%), and is denatured and rearranged to form a fibrous structure (5–8).

Soybean protein is the main raw material for plant-based products and is often blended with other plant proteins to prepare plant-based products with anisotropic structures (4). For example, the association of soybean protein with wheat protein in extruded products helps to enhance the formation of fibrous structures in soybean protein extrudates (5). High-moisture textured plant protein products currently cannot match the quality standard of meat in the food consumption market due to texture characteristic limitations (9–12). Researchers have attempted to improve their texture by adding polysaccharides (13). For example, soybean protein extrudates supplemented with 6% sodium alginate (SA) had the highest degree of organization and rehydration (14). Xanthan gum (XG) has shear dilution properties that enhance the mechanical strength of the product and is currently used in 3D printing (15). XG can modify the flow behaviour of the material and may fit in high moisture extrusion environments. Maltodextrins (MD) can influence the bound water content and cooking properties of extruded products (16). Thus, one way to improve the texture properties of extrudates is the addition of polysaccharides, which has been successful in plant-based products. However, existing studies have not identified differences in the effect of polysaccharides on the textural properties of high moisture extruded products of soybean and wheat proteins.

Polysaccharides have a good ability to bind water, providing processing properties that increase viscosity, limit water flow and improve mouthfeel (13). In polysaccharide-soybean protein composite extrusion processing systems, polysaccharides can bind and interact with more water in the form of hydrogen bonds, while increasing the consistency of the material during extrusion, leading to enhanced fibrillation of the extrudate (14–16). The interaction of different polysaccharides with plant proteins can further influence the molecular structure of high moisture extruded plant-based products, thus affecting the quality characteristics of plant-based products. As such, this study is aimed at assessing the impact of SA, XG, and MD on the physicochemical characteristics of plant protein tissue products. More importantly, the mechanism of polysaccharides to improve the quality of extrudates was investigated based on changes in material conformation and morphology in different extrusion zones. Textural properties, colour, water/oil absorption capacity, cooking properties, and specific mechanical energy of extrudates were examined. Low-field nuclear magnetic resonance was used to determine the moisture distribution. Characterization of the degree of polysaccharide-protein interaction, the shift in thermal properties of the samples in each zone and characterisation of the strength of protein molecular bonds by protein solubility were achieved. Finally, the microstructure of the material during extrusion processing was observed to further illustrate the feasibility of SA, XG, and MD in enhancing the quality of high-moisture extruded products, which is important for the future optimisation of the nutritional value of plant protein organised products and their application in mass production in the food industry.

## 2. Materials and methods

### 2.1. Materials

Soybean protein isolate (SPI) with 91.2% protein, 0.53% carbohydrate, and 0.65% fat (dry basis), and wheat gluten (WG) with 85.2% protein, 5.1% carbohydrate, and 1.0% fat (dry basis) were purchased from Jinlong Co., Ltd., (Harbin, China) and Wandefu Agricultural Development Co., Ltd., (Henan, China), respectively. Sodium alginate (SA), xanthan gum (XG), and maltodextrin (MD) were purchased from Qingdao Mingyue Seaweed Group Co., Ltd., (Qingdao, China) and Inner Mongolia Fufeng Biotechnology Co., Ltd. (Hohhot, China) and Xiwang group Co., Ltd. (Jinan, China), respectively. All chemical reagents were and of analytical grade and were purchased from Tianjin Tianli Chemical Reagent Co. (Tianjin, China).

### 2.2. Preparation of high-moisture extrusion samples

**FIGURE 1:**
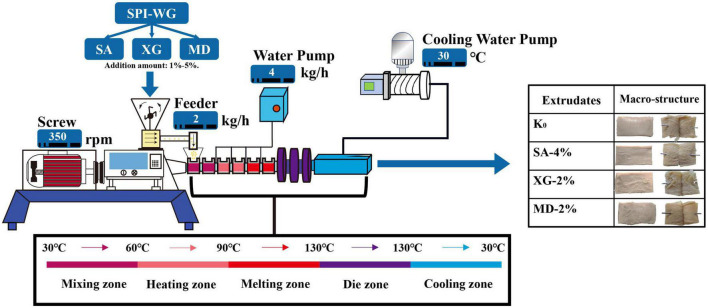
Schematic diagram of high-moisture extrusion cooking process of soybean-wheat protein with different amounts of sodium alginate (SA), xanthan gum (XG), and maltodextrin (MD) (including the division of sampling section and macro-structure of extrudates).

Extrusion experiments were carried out on a co-rotating twin-screw extruder (Clextralev-25 model, France) equipped with a screw diameter (25 mm) and L/D ratio (1:24). During the high moisture extrusion experiment, the screw speed was constant at 350 rpm. The feeder rate was constant at 2 kg/h. The water feed rate (4 kg/h) was adjusted so that the final moisture content of the product was 57%. SPI and WG with a ratio (70:30) (w/w, dry basis) as the base material, was mixed with SA, XG, and MD at 1, 2, 3, 4, and 5% (w/w, dry basis), respectively. The test group without the polysaccharides was used as a blank control (K_0_). The sleeve temperature was set at 30, 60, 90, 130, 130, and 130°C from the raw material input barrel as the starting point, with the cooling die head section being constant at 30°C (Figure 1). After waiting for the stabilisation of the extrudate, the die was disassembled within 5 min, the samples were collected and the extrusion test was stopped immediately. In particular, the samples were quickly collected from different sections of the barrel, sealed in a food vacuum package and stored at −18°C. Part of samples were freeze-dried (−60°C), ground, sieved through 100-mesh and used for further analysis.

### 2.3. Texture properties

The textural properties of the extruded samples were tested by referring to the research method by Peng et al. (11), with slight modifications. Samples were cut to 20 × 20 × 5 mm (L × W × H) and were set in full texture mode (TPA) in a Texture Analyzer (TA. XT Plus, Stable Micro System Co., Ltd., USA). The analysed parameters were hardness, chewiness, and elasticity. The pre-test speed, test speed, and post-test speed were 1 mm/s, the downward pressure ratio was 50% of the sample height and the interval was 4 s. The fibrous degree was determined by cutting the sample with a CKB probe at a speed of 1 mm/s which was calculated from the ratio of transverse and longitudinal shear forces in the extrusion direction (9). Tensile strength was measured with an A/TG probe by the stretching samples with a thickness of approximately 15 mm at a stretching rate of 1 mm/s [refer to the sample preparation diagram provided by Chen et al. (10) for the shape], and the maximum force and displacement at the time of sample rupture were recorded. All textural properties measurements were performed at 25°C.

### 2.4. Colour measurement

The colour of the extrudates (containing K_0_, SA-4%, XG-2%, and MD-2%) was measured using a CR-400 colorimeter (Konica Minolta Co., Japan) (at 25°C). After calibration with a standard white plate, five parallel measurements were made, and the maximum and minimum values were removed to obtain the L*, a*, and b* values of the samples, and the corresponding colour cards were drawn for analysis using Adobe Photoshop Software, referring to the method described by Lee et al. (17).

### 2.5. Cooking yield

The cooking yield of the extrudates (containing K_0_, SA-4%, XG-2%, and MD-2%) was carried out according to the method of Nisov et al. (18) with slight modifications. The extruded samples were cut into (2 × 2 × 2 cm, L × H × M) squares. The extruded samples were cooked in a stainless-steel pot at a constant temperature of 80°C for 20 min. After resting on a strainer for 15 min (at 25°C), the samples were weighed. The cooking yield of the extruded samples was calculated using Eq. 1:


(1) $$\mathrm{Cooking}\mathrm{yield}\left(\%\right)=\frac{m_{1}}{m_{0}}\times 100\%$$

where m_0_ was the initial weight of the extruded sample, m_1_ was weight of the extruded sample after cooking and filtering dry for 15 min.

### 2.6. Water absorption capacity (WAC) and oil absorption capacity (OAC)

Water absorption capacity (WAC) and oil absorption capacity (OAC) of the extrudates (containing K_0_, SA-4%, XG-2%, and MD-2%) were determined by referring to the method of Kantanen et al. (19). After being divided into strips (30 × 10 × 20 mm, L × H × M), the samples were dried in a 40°C oven for 24 h. The dried samples were put into 50 mL test tubes, mixed with 40 mL deionized water or canola oil, respectively, and hydrated in a water bath at 50°C for 12 h. Finally, the samples were air-dried on a sieve for 5 min (at 25°C). WAC or OAC of the samples was calculated according to Eq. 2:


(2) $$\mathrm{WAC}\mathrm{or}\mathrm{OAC}\left(\%\right)=\frac{{\text{M}}_{\text{b}}-M_{\text{a}}}{{\text{M}}_{\text{a}}}\times 100\%$$

where M_*a*_ was the weight of the sample after drying and M_*b*_ was the weight of the sample after hydration with deionized water or canola oil and drying.

### 2.7. Low-field nuclear magnetic resonance (LF-NMR)

To analyse the moisture distribution of the extrudates, each extrudate (containing K_0_, SA-4%, XG-2%, and MD-2%) was cut into small strips (5 × 10 × 10 mm thick, M × L × H) and placed in a 10 mm diameter NMR sample cuvette. The results of the moisture distribution of the extrudate were recorded on a low-field NMR analyser (Niumag Co., Ltd., Shanghai, China) (at 25°C). The transverse relaxation times (T_2b,21,22_) and peak ratio (P_2b,21,22_) of the samples were determined using a Carr-Purcell-Meiboom-Gill (CPMG) pulse sequence (6).

### 2.8. Specific mechanical energy (SME)

During high moisture extrusion, torque and sample yield per unit of time were assessed. SME was determined using Eq. 3 (20).


(3) $$\mathrm{SME}\,\left(\mathrm{kJ}\cdot {\text{kg}}^{-1}\right)=\frac{\mathrm{Screw}\mathrm{speed}\times \mathrm{Power}\left(\mathrm{kW}\right)\times \mathrm{Torque}\left(\%\right)}{\begin{array}{c} \mathrm{Maximum}\,\mathrm{screw}\,\mathrm{speed}\\ \times \mathrm{throughput}\,\left(\mathrm{kg}\cdot {\text{s}}^{-1}\right)\times 100 \end{array}}$$

where the extruder’s power was 15 kW, the maximum screw speed was 600 rpm and the actual screw speed was 350 rpm. The torque was recorded in real-time in the extruder control panel and the throughput was the output per second of extrudates (containing K_0_, SA-4%, XG-2%, and MD-2%).

### 2.9. Fourier transform infrared spectroscopy (FT-IR)

A Spectrum Two infrared spectrometer (PerkinElmer, USA) was used to perform Fourier transform infrared spectroscopy (FT-IR) to evaluate the secondary structure content of the extrudates (containing K_0_, SA-4%, XG-2%, and MD-2%) in the various extrusion zones. Utilising some adjustments to the approach outlined by Xia et al. (11), the freeze-dried, sieved sample powder (1.8 mg) was combined with KBr (180 mg) and then pressed. The blank substrate (air as a reference) was scanned 32 times at a wave number at 4,000 to 500 cm^–1^, 4 cm^–1^ resolution. The data were obtained by fitting the amide I band (1,700–1,600 cm^–1^) and using Fourier self-deconvolution and the second-order derivative fitting in the Peak Fit 4.12 software (SPSS Inc., Chicago, USA). The content of the secondary structure [α-helix (1,646–1,664 cm^–1^), β-sheet (1,615–1,637 and 1,682–1,700 cm^–1^), β-turns (1,664–1,681 cm^–1^), and random coil (1,637–1,645 cm^–1^) by the Amide I area were calculated with reference to Dou et al. (14).

### 2.10. The degree of grafting (DG)

Referring to the method provided by Chen et al. (10), 2 mg/mL of the extrudates (containing K_0_, SA-4%, XG-2%, and MD-2%) in each zone of the extrusion mixed with OPA (o-Phthalaldehyde) reagent [consisting of deioned water as the base solvent containing 0.8 g OPA, 500 mL sodium tetraborate solution (0.01 M, pH = 9.7), 50 mL sodium dodecyl sulphate (20% mass fraction), and 2 mL β-mercaptoethanol] at ratio (1:80) (v/v). The reaction was carried out in a water bath at 90°C for 5 min. The absorbance of the samples was measured at 340 nm using a UV-Vis spectrophotometer (ALPHA 1650, shanghai, China) using the raw material as a control, and the DG was calculated according to Eq. 4:


(4) $$\mathrm{DG}\left(\%\right)=\frac{{\text{A}}_{1}-{\text{A}}_{2}}{{\text{A}}_{1}}\times 100\%$$

where A_1_ was the absorbance of the raw material and A_2_ was the absorbance of the sample in each zone of the extrusion.

### 2.11. Thermal analysis

Differential scanning calorimetry (DSC) analysis of the extrudates (containing K_0_, SA-4%, XG-2%, and MD-2%) in each zone of the extrusion were achieved using DSC Q2000 (TA Instruments, New Castle, DE, USA) according to the method of Chen et al. (10). Sample (8 mg) was weighed and sealed in an aluminium tray, and the temperature range was set from 25 to 130°C. The temperature was ramped up at a heating rate of 5°C/min. The whole test was performed at constant nitrogen condition (50 mL/min) and the peak temperature (Tp) and enthalpy change (ΔH) of the samples were recorded.

### 2.12. Determination of protein solubility

The protein interactions during extrusion were reflected by studying the difference in protein solubility of the extrudates with different extrusion zones in specific chemical reagents. The study method by Fengqiujie Wang et al. (6), was utilised with slight modifications. Four specific extractants were used: (1) phosphate buffer (pH = 7.5, 0.1 mol/L); (2) 8.0 M urea in (1); (3) 0.05 M dithiothreitol in (1); (4) 0.05 M dithiothreitol and 8.0 M urea in (1). Sample (100 mg) was accurately weighed and dissolved in a centrifuge tube with 10 mL of extract, and then reacted in a water bath at 25°C for 2 h. The sample was centrifuged in a centrifuge (GL21M, Hunan, China) at 12,000 rpm for 20 min (at 25°C). According to the Bradford method, the supernatant was taken for the determination of soluble protein using a UV-visible spectrophotometer (ALPHA 1650, Shanghai, China) at 595 nm. The protein content of the sample was determined according to the Kjeldahl method. Protein solubility was calculated based on the ratio of soluble protein content in the supernatant to the protein of the sample.

### 2.13. Scanning electron microscopy (SEM)

The microstructural analysis of extrudates (containing K_0_, SA-4%, XG-2%, and MD-2%) from the extruded zones was carried out using the method by Dou et al. (14). The samples were cut into 3 × 10 × 2 mm (L × M × H) slices and placed in a beaker with 5 mL of glutaraldehyde (2.5% concentration; pH 7.2) and left to stand at 4°C for 1.5 h. The samples were rinsed three times with phosphate buffer (0.1 M; pH 7.2) for 10 min each. The samples were then dehydrated 3 times with ethanol and soaked in pure tert-butanol for 15 min. Finally, the samples were freeze-dried, coated and sprayed with gold. A scanning electron microscope (Zeiss supra-55, Germany) operating at an accelerating voltage of 15.0 kV was used to capture images of the surface morphology of the samples. The images were photographed at ×1.5 k.

### 2.14. Statistical analysis of data

The data were presented as mean ± standard deviation and all tests were performed in triplicate under the same conditions. Using analysis of variance, the statistical significance between all of the data was evaluated (ANOVA). At a level of (*p* < 0.05), Tukey s-b(K) was employed to assess the data for significant differences.

## 3. Results and discussion

### 3.1. Textural properties analysis

Important indexes for evaluating the quality of meat substitutes are texture characteristics (21). By calculating the ratio of the longitudinal shear force (N) to the transverse shear force (N) applied to the extrusion to obtain a degree of texturing, the formation of the fibrous structure was evaluated (22). As shown in Table 1, the addition of SA, XG and MD had a significant effect on the textural properties of the extrudates. Polysaccharides were added at different degrees to promote the formation of fibre structure and to reduce any negative effect of fibre structure. The addition of SA-4%, XG-2%, and MD-2% resulted in products with higher fibrous degrees than the control (1.11) and other polysaccharide groups of the same gradient, indicating the formation of a better fibre structure. Chen et al. (10) showed that tenderness of extrudates is usually associated with hardness, springiness and chewiness. Existing studies have not revealed the degree of hardness, springiness and chewiness required for meat analogues to achieve optimum quality (3). The highest fibrous degree of extrudate (2.1), hardness of 10,557.53 g, springiness enhancement to 0.92, and overall tensile strength (762.28 g) were exhibited by addition of SA at 4%. XG as a thickener and stabilizer can improve the viscosity of the system (23). Due to the increased viscosity of the system, addition of 2% XG resulted in the highest springiness (1.01) and tensile strength (634.28 g) which was higher than the control extrudate (467.94 g). However, as XG addition increased from 2 to 5%, the hardness, springiness and tensile strength of the extrudates were significantly reduced and therefore exhibited poorer fibrillation. This may be due to the fact that the use of excess XG inhibited the deformation of the raw material during extrusion, thus weakening the moulding ability of the samples (24). MD improved the adhesive properties of the product (25). A significant increase in hardness (10,258.47 g), springiness (0.92), and chewiness (9,091.6 g) were elicited by addition of 2% MD. In contrast, increase in MD had, a negative effect on the fibre structure formation of the extrudate, as reducing in hardness, chewiness and tensile properties of the extrudate occurred. A previous study revealed that addition of 10% straight-chain starch was detrimental to the formation of fibrillation of high moisture texturised pea protein (10).

**TABLE 1:** Texture characteristics, fibrous degree, tensile strength, and tensile distance of SPI-WG extrudates with different polysaccharides.

	Sample	Hardness (g)	Springiness	Chewiness (g)	Longitudinal shear force (N)	Transverse shear force (N)	Fibrous degree	Tensile strength (g)	Tensile distance (mm)
K_0_		7456.17 ± 436.24de	0.87 ± 0.02cd	6475.84 ± 307.03d	12.1 ± 0.23d	10.94 ± 0.09de	1.11 ± 0.03bc	467.94 ± 27.54ae	36.83 ± 3.00c
SA	1%	8053.86 ± 173.83e	0.90 ± 0.01e	7574.37 ± 267.53f	24.55 ± 0.21l	17.33 ± 0.04j	1.41 ± 0.02g	682.2 ± 14.27i	62.73 ± 1.51f
	2%	8797.70 ± 304.53fg	0.90 ± 0.01de	7082.93 ± 167.93ef	16.76 ± 0.24j	9.63 ± 0.25c	1.74 ± 0.04j	673.19 ± 22.52hi	78.32 ± 0.54g
	3%	10100.17 ± 114.49h	0.94 ± 0.01fgh	8845.61 ± 200.76g	17.74 ± 0.17k	9.85 ± 0.08c	1.8 ± 0.01k	691.65 ± 9.74i	57.77 ± 1.64e
	4%	10557.53 ± 442.37h	0.92 ± 0.01ef	8984.61 ± 253.03g	15.8 ± 0.07i	7.52 ± 0.12b	2.1 ± 0.03l	762.28 ± 14.55j	75.36 ± 3.19g
	5%	7025.36 ± 358.81d	0.75 ± 0.01a	6594.40 ± 87.84de	17.07 ± 0.12j	11.15 ± 0.25ef	1.53 ± 0.03i	316.56 ± 11.09c	28.29 ± 1.76b
XG	1%	6880.87 ± 182.38d	0.95 ± 0.01gh	5934.53 ± 151.88c	11.83 ± 0.07d	10.63 ± 0.20d	1.11 ± 0.01bc	617.56 ± 11.74g	61.53 ± 1.30ef
	2%	6187.61 ± 319.62c	1.01 ± 0.02i	6407.86 ± 511.27cd	14.69 ± 0.17h	9.96 ± 0.06c	1.47 ± 0.03h	634.28 ± 22.68gh	62.67 ± 1.77f
	3%	5697.14 ± 76.85c	0.96 ± 0.01h	4631.18 ± 234.99b	9.7 ± 0.12b	7.41 ± 0.15b	1.31 ± 0.01f	560.5 ± 27.77f	48.50 ± 1.37d
	4%	4626.13 ± 217.51b	0.89 ± 0.01cd	4142.96 ± 91.42b	12.76 ± 0.15e	11.64 ± 0.10g	1.10 ± 0.01bc	212.33 ± 15.00b	26.69 ± 0.93b
	5%	2862.47 ± 149.70a	0.93 ± 0.01efg	2374.30 ± 147.75a	11.46 ± 0.32c	10.71 ± 0.27d	1.07 ± 0.01b	139.84 ± 19.29	19.94 ± 0.72a
MD	1%	8383.52 ± 221.58fg	0.88 ± 0.02cd	7206.16 ± 128.97f	13.12 ± 0.10f	11.51 ± 0.39fg	1.14 ± 0.03cd	384.62 ± 35.24d	33.53 ± 1.38c
	2%	10258.47 ± 103.69h	0.92 ± 0.02ef	9091.60 ± 356.70g	15.51 ± 0.21i	12.86 ± 0.08i	1.21 ± 0.01e	433.8 ± 22.63e	37.15 ± 4.15c
	3%	13104.22 ± 485.05i	0.86 ± 0.02c	9686.36 ± 264.09h	13.59 ± 0.14g	11.51 ± 0.19fg	1.18 ± 0.02de	229.76 ± 16.05b	29.28 ± 1.11b
	4%	8093.87 ± 416.75f	0.81 ± 0.02b	6190.36 ± 365.56cd	6.01 ± 0.13a	6.39 ± 0.23a	0.94 ± 0.03a	231.25 ± 4.19b	26.40 ± 0.71b
	5%	7392.09 ± 328.78d	0.88 ± 0.02cd	6143.63 ± 54.93cd	11.23 ± 0.10c	12.15 ± 0.23h	0.92 ± 0.02a	125.01 ± 41.17a	18.83 ± 1.57a

Different small letters reveal significant differences between different samples separately (*P* < 0.05).

### 3.2. Physical properties in the extrudates

#### 3.2.1. Colour analysis

Colour is one of the key qualities of meat analogues and one of the important sensory criteria for consumers of meat analogues (3). As shown in Table 2, the measured L*, a*, and b* values were used to generate the corresponding colour swatches, which provide a visual representation of the colour of the extrudate (17). Extrudate colour is typically influenced by the Millard reaction (26), while in this study, addition of SA, XG, and MD had significant effect on the L* values. The brightest extrudate was obtained with the addition of 4% SA. This may be due to the good flowability of SA, which improved the material flow rate in the barrel and reduced the time of the Maillard reaction, thus improving the colour of the extrudate (14). Addition of both 2% XG and 2% MD resulted in darker extrudates compared to the control. This may be due to “pseudoplasticity” of XG, which affects the flow rate when the material is subjected to shear forces in different zones of the extrusion process (27). MD, by increasing the gelling properties of the material, may promote the aggregation of proteins and increase the viscosity of the system, thereby increasing the residence time of the material in the barrel (28). The longer the time that the material undergoes the Millard reaction inside the extruder, the increase in production of more dark material (significant increase in b* values), which reduces the brightness of the extrudate. In addition, polysaccharides not only change the colour of the extrudate, but can produce volatile compounds (e.g., pyrazine) through the Millard reaction, thereby giving the extrudate a new flavour (29).

**TABLE 2:** Colour value of extrudates with different polysaccharides.

Sample	L*	a*	b*	Color
K_0_	63.76 ± 0.46c	1.53 ± 0.01a	17.53 ± 0.29a	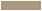
SA-4%	69.40 ± 0.41d	1.53 ± 0.05b	18.85 ± 0.42b	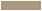
XG-2%	62.03 ± 0.82b	1.53 ± 0.08a	20.03 ± 0.30c	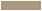
MD-2%	59.02 ± 0.58a	1.53 ± 0.13c	20.57 ± 0.24c	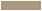

Different small letters reveal significant differences between different samples separately (*P* < 0.05).

#### 3.2.2. Cooking yield

**FIGURE 2:**
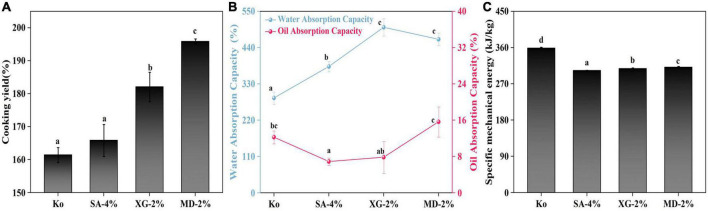
Effect of different contents of sodium alginate (SA), xanthan gum (XG), and maltodextrin (MD) of the extrudates on the **(A)** Cooking yield, **(B)** Water absorption capacity and Oil absorption capacity, and **(C)** Specific mechanical energy. Different letters imply significant differences between different samples (*P* < 0.05).

The difference in water absorption of the extrudate under high-temperature cooking can directly affect the tenderness of the extrudate (30). As shown in Figure 2A, the addition of SA, XG and MD enhanced the steaming properties of the extrudates. The order of the ability of polysaccharides to enhance steaming properties was MD-2% > XG-2% > SA-4%. This may be due to the fact that these three polysaccharide molecules possess a large number of hydroxyl functional groups, which can form hydrogen bonds with water molecules and enhance the hydrogen bonding content between proteins. At the same time, this may promote the formation of protein-polysaccharide complexes and enhancing the water absorption capacity of the extrudates, which can absorb more water after steaming (13). It was reported that MD showed elongation of polymer chains, increased water affinity rand enhanced hygroscopicity at high moisture humidity (31). Therefore, the addition of 2% MD promoted better water binding of extrudates and reduced cooking moisture loss. This prevents the extruded product from losing a lot of water during the cooking process and prevents excessive loss of nutrients. In addition, the above results also suggest that the increased cooking yield of the extrudate was due to a reduction in moisture loss. This suggests that the addition of different polysaccharides altered the extrudate structure and was vital in understanding the differences in cooking properties (32).

#### 3.2.3. Water and oil absorption capacities (WAC and OAC)

Water absorption capacity indicates the extent to which the extrudate can interact with water and is influenced by the mechanical properties of the extrudate, hydrophilic groups, number of polar sites, etc (33). Addition of the three polysaccharides significantly increased the water absorption capacity of the extrudates compared to the control (Figure 2B). Among them, the water absorption capacity of XG-2% was increased by 174.64%. This may be because the polysaccharides are rich in hydrophilic groups and can form more hydrogen bonds to retain the water molecules (34). Therefore, addition of SA, XG, and MD was able to enhance the water absorption capacity of the extrudates. This property may offer the possibility of adding water-soluble nutrients to food processing.

Oil absorption capacity depends mainly on the number of hydrophobic groups on the extrudate surface, reflecting the extent to which the extrudate can bind to oil (12). In contrast to the water absorption capacity results, the oil absorption capacity decreased significantly with addition of polysaccharides, reaching a minimum value of 6.86% at a SA addition of 4%. When extruded at high moisture, the raw material interacts more with water, forming a fibrous structure through protein denaturation and aggregation, resulting in a large number of hydrophobic groups being masked inside the molecule which hinders oil binding ability (35).

#### 3.2.4. Specific mechanical energy (SME)

Specific mechanical energy is considered as the magnitude of mechanical energy absorbed by the extrudate and can reflect the flow behaviour of the feedstock in the extruder (17). As seen in Figure 2C, addition of SA/XG/MD to SPI-WG significantly decreased SME in the order: SA-4% > XG-2% > MD-2%. This indicates that the addition of polysaccharides assisted in decreasing viscosity of the extruded material during the extrusion process and effectively promoted faster flow of the extruded material. The addition of polysaccharides was able to reduce energy consumption during high-moisture extrusion of pea protein, while the lower SME enhanced the fibrous structure of the soybean protein extrudate (36). For example, compared to the control, the SME of XG-2% decreased from 356.63 to 307.92 kJ/kg, while the fibrous degree increased from 1.11 to 1.47. This finding is consistent with (37), who revealed a gradual decrease in SME as the fibrous degree of high-moisture extruded soybean protein products increased.

#### 3.2.5. Water distribution

**FIGURE 3:**
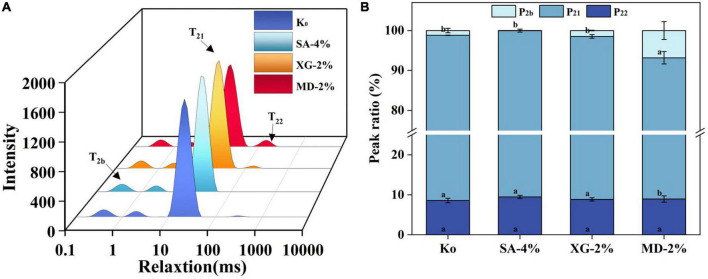
Effect of different contents of sodium alginate (SA), xanthan gum (XG), and maltodextrin (MD) of the extrudates on **(A)** the transverse relaxation times (T_2b,21,22_), **(B)** the peak ratio (P_2b,21,23_). Different letters imply significant differences between different samples (*P* < 0.05).

Low-field nuclear magnetic resonance is a powerful tool to assess the state and distribution of water in food (38). T_2_ relaxation time is the time taken to reach dynamic equilibrium between protons through energy interchange (39). Shorter or longer lateral relaxation times correspond to lower or higher degrees of freedom, respectively. Whereas, the most strongly bound to the macromolecules in the sample was bound water (T_2b_), followed by moderately bound fixed water (T_21_) and weakly bound free water (T_22_) (6). The water distribution of the SPI-WG extrudate with addition of SA, XG and MD was measured using LF-NMR. As seen in Figure 3A, the bound water in SA-4%, XG-2%, and MD-2% were all higher than the control, indicating that more water molecules were present inside the polysaccharide-protein extrudate. Previous studies have shown that addition of polysaccharides enhances hydrogen bonding between proteins and restricts the mobility of water molecules (40). The control group showed the lowest T_21_ (peak area of 9,217.96), which implies that addition of SA (peak area of 9,349.34), XG and MD (peak area of 9,297.18) immobilised more water during the extrusion. Among them, XG had the largest T_21_ (peak area of 9,972.45), which could be due to the strong gelation properties displayed by XG after extrusion processing, which prompted the protein-polysaccharide complex to encapsulate more water (41). Interestingly, addition of 2% MD had the largest percentage of T_22_ of 6.82% (Figure 3B), indicating that MD-2% had a stronger adsorption capacity for free water. This coincides with the corresponding high steaming property possessed by MD-2% (Figure 2A).

### 3.3. Fourier transform infrared spectroscopy (FT-IR)

**FIGURE 4:**
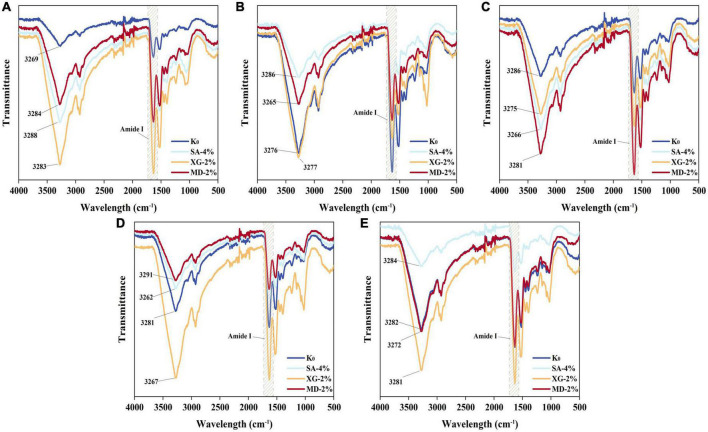
Fourier transform infrared spectroscopy (FT-IR) spectra for extrudates in different zones. **(A)** Mixing zone, **(B)** Heating zone, **(C)** Melting zone, **(D)** Die zone, and **(E)** Cooling zone.

Polysaccharides contains hydroxyl functional groups and a large number of inter- or intra-molecular hydrogen bonds, enabling the indirect detection of information on the changes in the strength of hydrogen bonds between samples by FT-IR (42). As shown in Figure 4, the sample shows a wider band at 3,600–3,200 cm^–1^, which is caused by the high-frequency stretching vibration of intermolecular O-H. The lower wave number indicates that the hydrogen bonding interaction between components was enhanced (43). In the mixing zone where the raw material has not yet been in contact with water, the experimental group containing polysaccharides showed a red shift in the 3,600–3,200 cm^–1^ region. This indicates that the anhydrous extrusion did not form new hydrogen bonds after addition of polysaccharides, but weakened the hydrogen bonds in the protein network. In the heating zone, addition of water promoted the protein-polysaccharide mixture to react at different degrees, with the lowest band wave number (3,265 cm^–1^) at MD-2%, implying that more hydrogen bonds may have been formed. As the material entered the melting zone, the material melted and the molecular structure unfolded, increasing the surface area in contact with water, and the bands all moved to shorter wavelengths, at which time the hydrogen bonding interaction in the polysaccharide test group was significantly enhanced. It is noteworthy that in the mixing zone-die region, XG-2% and SA-2% consistently enhanced the hydrogen bonding interactions between the components (3,283–3,267 and 3,288–3,262 cm^–1^). This may be caused by the pseudoplasticity of XG (shear thinning under high shear) and the strong water absorption capacity of SA (capable of adsorbing about 200–300 times its own volume of water) (40, 44). In the heating zone-die region, MD-2% weakened the hydrogen bonding interaction between components (3,265–3,291 cm^–1^). This was probably due to the reaction of MD with protein which formed a certain water-insoluble polymer, which hindered the partial binding of the sample to water. In the cooling zone, with decrease in temperature, low shear and the cessation of water input, only the 3,291 cm^–1^ band of MD-2% shifted toward 3,272 cm^–1^. It is presumed that MD-2% binded with more water (corresponding to the LF-NMR results in Figure 3A), which is likely to be free water.

**FIGURE 5:**
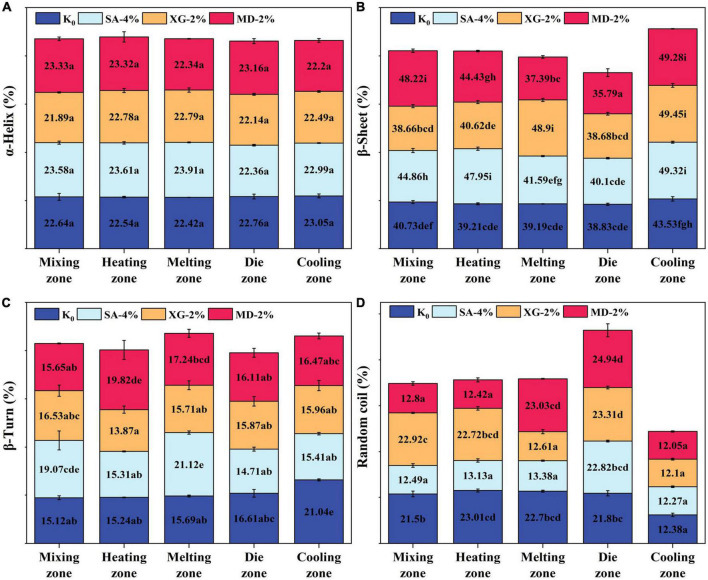
Relative proportion changes of protein secondary structures for extrudates of different zones. **(A)** α-Helix, **(B)** β-Sheet, **(C)** β-Turn, and **(D)** Random coil. Different letters imply significant differences between different samples (*P* < 0.05).

The amide I band (1,600–1,700 cm^–1^) of the FT-IR spectrum correlates with the vibrational state of the protein chemical bonds (e.g., C=O, C–N), reflecting the variation of the secondary structure of the samples in different extrusion zones (Figure 5). In the mixing-cooling zone, the α-helix structure of the samples remained unchanged, mainly with transitions between β-sheet, β-turn and random coil structures. The β-sheet content of all samples showed a trend of first increasing, then decreasing and finally increasing. In the mixing-melting zone, the protein was initially denatured and the chains were stretched gradually forming a certain rigid structure (2). Although SA-4% and MD-2% showed a reduced content of β-sheet in the melting zone, XG-2% showed a higher content of β-sheet, indicating that XG-2% had better thermal stability. In the die zone, the high temperature disrupted the chemical bonds that maintained the conformation of the mixture. This elevated the surface hydrophobicity of the mixture, thus reducing the protein-water interaction and disrupting the ordered structure (8). In the cooling zone, more β-sheet structures are formed through transitions between the secondary structure components, promoting a more ordered structure formation in the extrudate. Compared with the control group, β-sheet structures of SA-4%, XG-2%, and MD-2% were significantly increased from 44.86, 38.66, and 48.22% to 49.32, 49.45, and 49.28%, respectively. Proteins underwent cross-linking and formation of aggregates which correlated with changes in the β-turn around the structure (10). In particular, the β-turn angle content of SA-4% increased significantly in the melting zone, from 19.07 to 21.12%, and decreased significantly in the cooling zone from 21.12 to 15.41%. This indicates that SA-4% underwent aggregation initially, followed by rearrangement and re-aggregation, and similar trends were observed from XG-2% and MD-2%. SA-4%, XG-2%, and MD-2% were found to have the highest content of random coil in the die zone. It was reported that the samples were extruded at high temperatures (150°C) and observed an increase in the content of random coil accompanied by a decrease in the content of β-sheet structures, which resulted in a disordered arrangement of protein molecules (14). Thus, it is evident that the enhancement of the extruded material ground properties may be due to the binding and transformation behaviour of SA, XG, and MD with proteins in the mixing-cooling zone. This mainly promoted the unfolding of the protein structure of the SPI-WG mixture, improved thermal stability and enhanced the protein aggregation ability, as reflected in the increase of β-sheet structure and the decrease of the β-turn structure, leading to the final formation of extrudates with a dense and rigid fibrous structure.

### 3.4. The degree of grafting (DG)

The degree of grafting can reflect the degree of the Maillard reaction in samples at different extrusion sections (45). The effect of SA, XG, and MD on the grafting degree of SPI-WG extrudate are shown in Table 3. In the mixing zone, addition of SA, XG, and MD significantly improved the grafting degree of the extrudate. This phenomenon is consistent with the bandwidth at 3,600–3,200 cm^–1^ observed by infrared spectroscopy. The carboxyl groups in the polysaccharide molecules interacted with the free amino group of protein through the Maillard reaction, which weakened the intramolecular and intermolecular hydrogen bond interaction (46). In the heating zone, SA-4%, XG-2%, and MD-2% showed higher grafting degrees, which may be caused by the binding of the hydrogen bonds with the initially denatured and stretched SPI-WG protein molecular chains. In the melting zone, XG-2% and MD-2% further consume free amino groups, resulting in higher grafting degree. At the same time, adding 4% SA provided favourable conditions for the subsequent dissociation and extension of SPI-WG, which shows that the grafting degree (38.02%) in the die zone was significantly improved. Compared with SA-4% (38.02%) and XG-2% (33.1%), MD-2% showed lower grafting degree (31.34%) in the die zone. The results indicated that MD-2% was disordered at high temperature, which was not conducive for protein aggregation and was consistent with the change in the protein secondary structure (Figure 5). In the cooling zone, the grafting degree of XG-2% increased by 24.8% compared with the die zone. The grafting degree of the control group and SA-4% decreased by 31.15 and 22.12% compared with the die zone, respectively, while the grafting degree of MD-2% was almost the same as that of the die zone. This indicates that XG can promote protein aggregation and crosslinking in the cooling zone, and produce more ordered fibre structures. However, the decrease in grafting degree of the control group and SA-4% may be due to decrease in the Maillard reaction degree of samples under the of low temperature, anhydrous environmental conditions and elongation in the cooling section (10). Thus, the colour of the final extrudate was brighter (Table 3).

**TABLE 3:** Degree of grafting and thermal properties of extrudates in different zones, in table, respectively.

Extrusion zones	Samples	Degree of grafting (%)	Tp (°C)	Δ H (J/g)
Mixing zone	K_0_	12.6 ± 0.76a	82.31 ± 0.49a	0.33 ± 0.4ab
	SA-4%	15.22 ± 1.67abc	84.77 ± 2.62ab	0.64 ± 0.29ab
	XG-2%	16.97 ± 1.91bc	93.37 ± 13.07abcd	0.3 ± 0.11ab
	MD-2%	14.36 ± 1.1ab	91.44 ± 2.07abcd	0.7 ± 0.32ab
Heating zone	K_0_	32.23 ± 0.55ghi	88.37 ± 0.95abc	0.65 ± 0.04ab
	SA-4%	37.63 ± 2.45jkl	102.64 ± 21.19bcd	0.71 ± 0.74ab
	XG-2%	18.02 ± 2.43c	84.81 ± 4.5ab	0.63 ± 0.13ab
	MD-2%	26.6 ± 1.72def	104.18 ± 2.36cd	0.54 ± 0.66ab
Melting zone	K_0_	33.11 ± 0.53hi	82.85 ± 10.01a	0.24 ± 0.23a
	SA-4%	27.16 ± 1.22ef	92.6 ± 0.74abcd	1.1 ± 0.04b
	XG-2%	23.28 ± 1.84d	86.03 ± 1.51abc	0.66 ± 0ab
	MD-2%	39.73 ± 2.27lm	91.26 ± 0.71abc	0.8 ± 0.33ab
Die zone	K_0_	35.35 ± 2.09ijk	90.29 ± 16.89abc	0.51 ± 0.32ab
	SA-4%	38.02 ± 1.52klm	89.04 ± 4.99abc	0.7 ± 0.66ab
	XG-2%	33.1 ± 1.48hi	86.99 ± 0.2abc	0.71 ± 0.18ab
	MD-2%	31.34 ± 1.58gh	95.03 ± 3.62abcd	0.54 ± 0.03ab
Cooling zone	K_0_	24.34 ± 0.94de	85.72 ± 2.63abc	0.49 ± 0.07ab
	SA-4%	29.61 ± 1.01fg	109.92 ± 10.83d	0.77 ± 0.45ab
	XG-2%	41.31 ± 2.24m	103.52 ± 0.23bcd	1.04 ± 0.08ab
	MD-2%	34.5 ± 0.28hij	88.56 ± 0.91abc	0.45 ± 0.1ab

Different small letters reveal significant differences between different samples separately (*P* < 0.05).

### 3.5. Thermal properties

**FIGURE 6:**
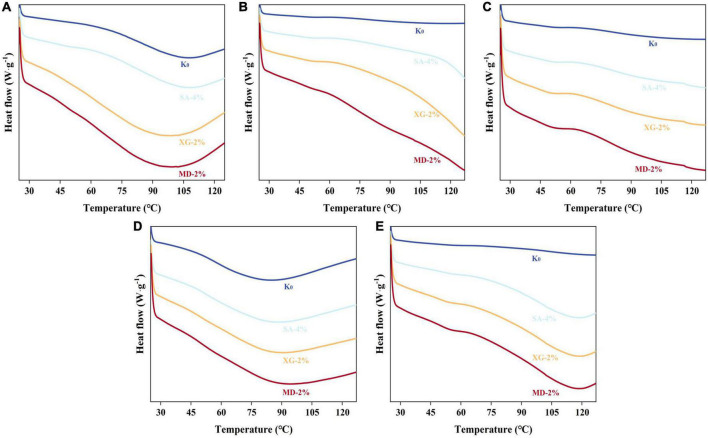
Differential scanning calorimetry characteristics of extrudates in different zones. **(A)** Mixing zone, **(B)** Heating zone, **(C)** Melting zone, **(D)** Die zone, and **(E)** Cooling zone.

The endothermic peak of a DSC endothermic curve mainly reflects the denaturation temperature (Td) of protein and the energy change (ΔH) during heating (47). The denaturation of raw material protein is one of the important factors to form a fibre structure, and the change of denaturation temperature may affect the product characteristics of an extrudate (48). As shown in Figure 6, the endothermic peak mainly appeared at 80∼110°C, which was due to the energy conversion of raw materials caused by thermochemical reactions such as protein thermal crosslinking and the Maillard reaction between the polysaccharides and protein. In the mixing zone, the addition of SA, XG, and MD significantly increased the Td and ΔH of the mixture. This indicates that polysaccharide was beneficial to increase the thermal stability and expand the structure of the mixture. In the heating-die zone, on one hand, the increase of processing temperature (60∼130°C) led to the unfolding of protein. This promoted interaction between the exposed molecular sites on the protein surface and polysaccharide and water, and formed a stable hydrophobic aggregate, which led to decrease in denaturation temperature of the mixture (10). On the other hand, a large amount of water was tightly bound to the protein-polysaccharide in the mixture, and a new molecular bond energy crosslinking occurred first and ΔH increased. Then, the protein formed a melt at high temperature (130°C). Due to thermodynamic incompatibility and lubrication by water, the friction and mechanical energy decreases, which means that the conversion of heat energy in the system is reduced, the intermolecular force is destroyed, and the crosslinking between protein-protein and protein-polysaccharide is weakened (49). Among them, SA-4% in the heating zone and melting zone showed higher thermal transition temperature and ΔH because it binded with more water molecules, which implied that the mixture in this zone had higher compactness. In the cooling zone, especially in the XG-2% experimental group, ΔH increased by 92.59%, which means that the protein recross-linked and formed new molecular bonds to stabilise the conformation. However, MD-2% showed lower ΔH, which may be due to the tight binding of MD with some water, which reduced the hydration of protein. This decreased denaturation temperature (88.56°C) and reduced the energy needed for denaturation of protein components.

### 3.6. The protein solubility analysis

**FIGURE 7:**
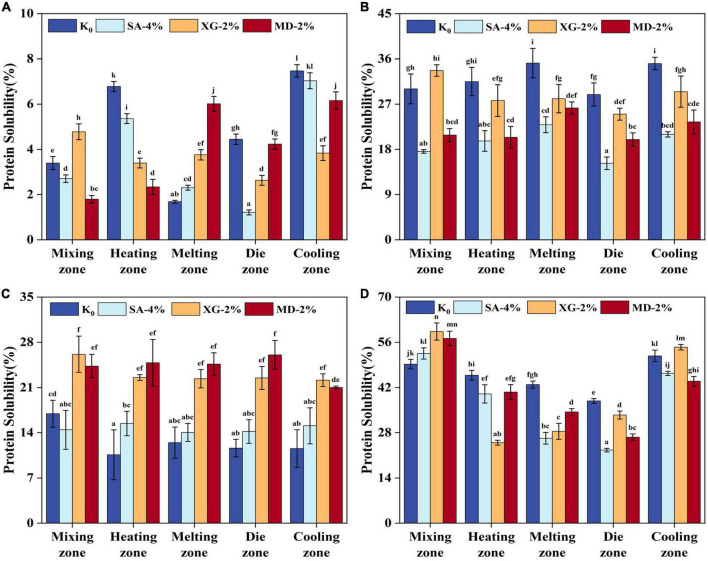
Protein solubility of the extrudates in different zones (Mixing zone, Heating zone, Melting zone, Die zone, and Cooling zone) induced by extracting solutions: **(A)** Phosphate buffer (pH = 7.5, 0.1 mol/L). **(B)** 8.0 M urea in phosphate buffer (pH = 7.5, 0.1 mol/L). **(C)** 0.05 M dithiothreitol in phosphate buffer (pH = 7.5, 0.1 mol/L). **(D)** 8.0 M urea and 0.05 M dithiothreitol in phosphate buffer (pH = 7.5, 0.1 mol/L). Different letters imply significant differences between different samples separately (*P* < 0.05).

The effect of different cross-linking bonds between proteins on the formation of fibre structure was analysed by measuring the protein solubility of the samples in different extrusion zones. As shown in Figure 7, the protein solubility of the samples ranged from 1.21 to 59.33%. The phosphate buffer was able to extract natural proteins. All squeezed samples showed the lowest protein solubility in phosphate buffer (Figure 7A). This indicates that most of the proteins underwent thermal denaturation under high moisture extrusion conditions, which changed the original structure of the protein and increased the degree of protein aggregation, thus exhibiting the lowest solubility (8). Urea is one of the reagents used to break hydrogen bonds and hydrophobic interactions, while dithiothreitol is one of the reducing agents used to cleave the disulphide bonds of proteins. Both reagents interacted *via* non-covalent and covalent interactions between samples, respectively (50). The results showed that the order of the proportional size of the chemically cross-linked bonds maintained in the extrudates was: non-covalent interactions > covalent interactions, which is consistent with that reported by (6). It can be seen that the protein structure of the extruded samples is mainly maintained by hydrogen bonds and hydrophobic interactions, followed by disulphide bonds. The solubility of proteins gradually increased in the mixing-melting zone, gradually decreased in the die zone, and finally rebounded in the cooling zone. This indicates that the rupture and reorganisation of protein molecular bonds occurred during the extrusion process, where the covalent and non-covalent interactions of proteins were reduced under high moisture, temperature and shear conditions leading to stabilised protein conformation, enhanced protein cross-linking and formed new protein molecular bonds when subjected to low temperature and low shear (2). Moreover, in urea (Figure 7B), different zones of SA-4%, XG-2%, and MD-2% showed lower proteolysis compared to the control, while in dithiothreitol (Figure 7C), different zones of SA-4%, XG-2%, and MD-2% showed higher proteolysis. This indicates that the different extruded zones had significant effect on the non-covalent interactions of the samples. Previously, researchers reported that the addition of polysaccharides reduced the viscosity of the melt, limited the contact area between water and protein, promoted polymerisation between protein-protein and protein-polysaccharide, and generated more stable disulphide bonds internally (14). The highest protein solubility of the samples was observed in Figure 7D in the presence of urea and dithiothreitol, suggesting that the extrudates were mainly composed of covalent and non-covalent interactions together to maintain the stability of the oriented protein structure. The overall decreasing then increasing trend further indicates that the cooling zone is the key zone for fibre orientation formation. The above results confirmed that SA, XG and MD affected the protein network structure of the extrudate by altering the chemical cross-linking bonds between the proteins. Although the addition of 2% XG improved the covalent and non-covalent interactions of aggregates formed in the die-cooling zone by about 62.79%, the excessive promotion of protein aggregation was not beneficial to the fibre structure orientation based on the results of extrudate ground analysis. On the contrary, SA-4% better balanced the conversion of chemical cross-linking bonds between samples. In the mixing-cooling zone, the disulphide bonding of SA-4% was stabilised at about 14.62%, and the hydrogen bonding and hydrophobic interactions were stabilised at about 19.28%, which promoted the samples to finally form the highest fibrous degree (2.1).

### 3.7. Microstructure of the extrudates

**FIGURE 8:**
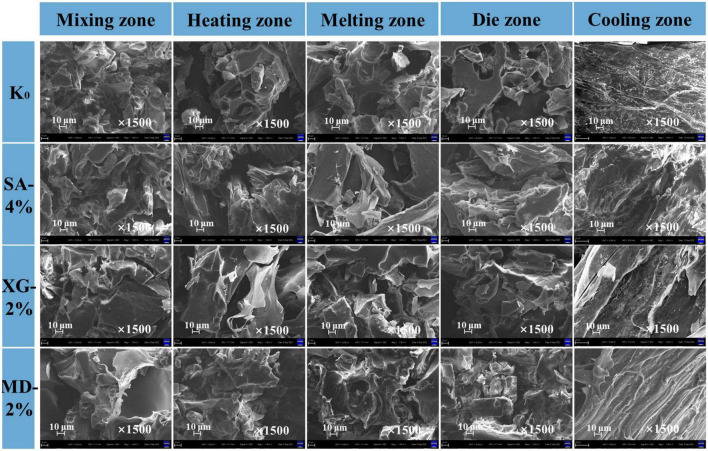
Scanning electron microscopy (SEM) images of the extrudates from different zones.

The formation of fibrous structures by raw material extrusion is usually considered a black box process, and scanning electron microscopy was performed on samples from different extrusion zones to further understand the effect of adding 4% SA, 2% XG, and 2% MD on the microstructure of SPI-WG extrudates (Figure 8). The mixing zone showed initial changes in protein structure, where some block structure formation was observed for SA-4%, XG-2%, and MD-2%. In the heating-die zone, expansion occurred due to the absorption of water by the protein particles (14). The porous structure was observed in the control group, but the critical fibrous structure was not shown as a whole. In addition, the experimental group with polysaccharide formed part of the block structure and improved part of the protein structure. Among them, SA-4% clearly showed some fibre orientation in the die zone. XG-2% showed the most disordered structure in the die zone, indicating that it significantly improved the flowability of the mixture. In the cooling zone, the structure of the extrudate changed significantly, and the appearance of a fibre network structure was observed. As reported by (2), the key region for protein formation of the anisotropic structure of the extrudate is in the cooling zone where the relatively low shear force contributes to laminar flow. The test group with addition of 4% SA, 2% XG, and 2% MD showed more fibrillar structures compared to the smooth structure of the control group. The SA-4% group had a distinct fibrillar structure and tight gel-like formation. The XG-2% group showed local agglomeration in the cross section. The MD-2% group had a coarser strip fibre morphology, consistent orientation and a flatter interface but the lower layer showed the presence of a rough porous structure in the lower layer. In comparison, addition of 4% SA was beneficial to the structural stability of different extruded zones, addition of 2% XG was beneficial to the flowability of the mixture at high temperature and aggregation at low temperature, and addition of 2% MD formed a coarse fibrous filament structure similar to that of meat. This indicates that SA, XG, and MD showed differences in properties in the extrusion environment and are advantageous in modifying the fibrous structure of the extrudate.

## 4. Conclusion

In this study, the structure and physiochemical properties of SPI-WG extrudates with the addition of SA, XG, and MD under high moisture extrusion conditions were comprehensively investigated. The results showed that the addition of SA, XG and MD provided the possibility to improve the formation of anisotropic fibre structure of SPI-WG mixtures, and the textural properties of the extrudates (e.g., fibrous degree) could be significantly improved. XG-2% has the strongest water absorption capacities (501.57%). MD-2% had the largest percentage of free water (6.82%). SA-4% has the highest moderately bound fixed water (peak area of 9349.34). The hydrophilicity of SA, XG, and MD helped to regulate the moisture distribution and gelling ability of the extrudates, which may be related to the transformation of the secondary structure of proteins. The degree of protein folding and flow ability determined the fibrous structure formation of the extrudate. Controlling the transformation of chemical cross-linked bonds of the feedstock in different extrusion zones and critically understanding the breaking and reorganization behaviour of chemical bonds through the melting zone-cooling zone are important factors for the formation of protein-polysaccharide blends into high-moisture extrudates with a characteristic three-dimensional protein network. In addition, by enhancing the thermal stability of the feedstock, higher energy conversion led to more dense protein network formation. SA-4% resulted in a desirable fibre structure (to enhance 89% than K_0_) by stabilizing intermolecular interactions and enhancing the thermal transition temperature of the system. Findings from this study have demonstrated an alternative means of conferring beneficial functional and nutritional properties to high-moisture extruded soybean protein-wheat protein thereby enhancing quality of high-moisture extruded plant protein products.

## Data availability statement

The original contributions presented in this study are included in the article/supplementary material, further inquiries can be directed to the corresponding authors.

## Author contributions

FW and XZ contributed to the conception and design of the study. FW performed the statistical analysis and wrote the first draft of the manuscript. YG helped with the experiment. All authors contributed to the manuscript revision, read, and approved the submitted version.
